# Juvenile catamenial pneumothorax: institutional report and review

**DOI:** 10.1186/s13019-015-0289-7

**Published:** 2015-06-13

**Authors:** Takashi Inoue, Masayuki Chida, Hirohisa Inaba, Motohiko Tamura, Satoru Kobayashi, Tetsu Sado

**Affiliations:** 1 Department of General Thoracic Surgery, Dokkyo Medical University, 880 Kitakobayashi, Mibu, Tochigi, 321-0293 Japan; 2 Department of General Thoracic Surgery, Shizuoka Red Cross Hospital, Shizuoka, Japan

**Keywords:** Catamenial pneumothorax, Endometriosis, Thoracic endometriosis syndrome, Pneumothorax

## Abstract

**Background:**

Catamenial pneumothorax (CP) is a type of spontaneous pneumothorax due to thoracic endometriosis occurring in reproductive women, and usually involves the right side of the thorax showing diaphragm lesions. For the present study, we defined juvenile CP (JCP) as patients with CP who were 19 years old and younger. Institutional findings and a systematic literature review are presented.

**Methods:**

We retrospectively enrolled all patients with CP treated at our institutions from January 2002 to June 2013. In addition, we conducted a search of medical literature published using the PubMed and Japanese Ichushi databases with “catamenial pneumothorax” as the search term.

**Results:**

Thirteen female patients with CP, 1 on the left side, were treated at our institutions. The patient with left-side CP was classified as JCP, while that was also identified in 29 of 451 CPs reported in our literature review. Pneumothorax occurred more frequently on the left side in JCP as compared to usual CP (p<0.01). There was a significantly lower ratio of JCP cases with diaphragm lesions as compared to usual CP (p<0.01).

**Conclusion:**

Significant laterality was not seen in JCP patients and fewer had diaphragm lesions as compared to usual CP. JCP may be a new entity of CP.

## Background

Catamenial pneumothorax (CP), an entity of thoracic endometriosis syndrome, was initially reported by Mauere in 1958 [1] and shown to comprise 3–6 % of female pneumothorax cases [2]. Several theories have been proposed to explain development of thoracic endometriosis including coelomic metaplasia, lymphatic or hematogenous embolization of an endometrial cluster, and retrograde menstruation with subsequent transperitoneal-transdiaphragmatic migration of endometrial tissue [3]. However, none of those can adequately explain all of the clinical manifestations of CP, whereas CP is unilateral or right-sided in nearly all cases, with diaphragmatic abnormalities such as perforation or endometrial deposits of tendinous portions common findings, indicating that transdiaphragmatic migration occurs in most affected individuals.

CP occurs in women during the reproductive years, mostly between 30 and 40 years old, while occurrence in females younger than 20 is rare. Based on our experience with a case of CP in a teenager, who had left-side pneumothorax without diaphragmatic lesion, we speculated that the mechanism of CP in younger individuals is different from that in older CP cases in regards to transdiaphragmatic migration. Therefore, for the present study we defined CP occurring in patients younger than 20 years old as juvenile CP (JCP), and retrospectively analyzed case records and also conducted a search of relevant medical literature.

## Methods

Thirteen CP patients who underwent surgery from January 2002 to June 2013 at our institutions were investigated in a retrospective manner. CP was defined as pneumothorax in a reproductive-aged female with endometrial tissue shown in a resected specimen or diaphragmatic lesion, such as blueberry spots or perforation. The Dokkyo Medical University Hospital Ethics Committee approved the study and waived the need for patient consent for analysis of the results.

### Search strategy

We also conducted a search of related medical literature using the PubMed and Japanese Ichushi databases using the term “catamenial pneumothorax” appearing in studies published between January 1974 and June 2013. Meeting abstracts were also included in that review. For publications that included updates and additions to prior publications, the most current was included in our analysis. Reports that did not designate the laterality of CP were excluded.

### Definition

We speculated that the mechanism of CP occurring in younger individuals is different from that in older cases in regard to transdiaphragmatic migration. Thus, we defined CP occurring at age of 19 years and younger as JCP, while that appearing at 20 years old and older as usual CP (UCP).

### Statistics

Analysis with a contingency table was performed using a chi-square test with Yate’s correction, while relative risk with a 95 % confidence interval was calculated using InStat 3 (GraphPad Software, Inc.). Statistical significance was considered with p values <0.05.

## Results

### CP patients treated at our institutions

The characteristics of patients with CP treated at our institutions are summarized in Table 1. Their mean age was 40.2 ± 9.0 years (range 18–53 years). Eleven cases were right-sided, 1 left-sided, and 1 bilateral. Twelve of our 13 patients had a diaphragmatic lesion, all of which were right-sided. Case 10 was left-sided JCP and without a diaphragmatic lesion.

**Table 1:** Characteristics of patients with catamenial pneumothorax

Case	Age	Laterality	Diaphragmatic lesion	Endometrial tissue*
1	41	Right	+	-
2	53	Right	+	-
3	47	Bilateral	+	-
4	29	Right	+	+
5	37	Right	+	+
6	44	Right	+	+
7	50	Right	+	-
8	44	Right	+	+
9	43	Right	+	-
10	18	Left	-	+
11	33	Right	+	-
12	45	Right	+	-
13	38	Right	+	-

*Pathologically proven endometrial tissue in surgical specimen

**Fig. 1:**
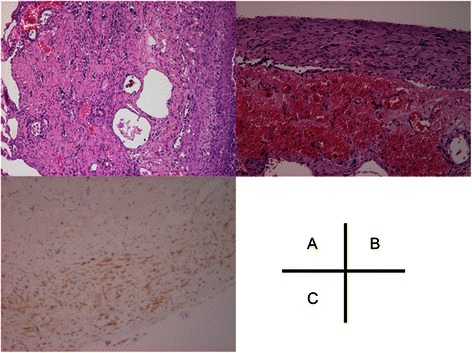
**a**: Endometrial, stroma, and gland tissues around the bulla (H&E, ×40). **b**: Fresh and old hemorrhage with hemosiderin-laden macrophages (H&E, ×200). **c**: Immunohistochemical staining of stroma cells showing a positive reaction for CD10 (×200)

Case 10 was an 18 year-old female who had dyspnea on effort after menstruation. Chest radiography revealed a left pneumothorax and chest computed tomography showed a small bulla in the apex of the left upper lobe. Closed chest tube drainage was performed, but failed to obtain expansion of the lung due to continuous air leakage. A thoracoscopic bullectomy was performed 5 days after the next menstruation. Several red spots were observed on the visceral pleura, though there was no evidence of a diaphragmatic lesion shown by blueberry spots or perforation. Pathological examination findings revealed the existence of CD10-positive endometrial tissue, stroma, and glands around the bulla, accompanied by fresh and old hemorrhage with hemosiderin-laden macrophages (Fig. 1). The post-operative course was uneventful and the patient was discharged 3 days after the operation. She then underwent hormonal therapy and no recurrence of pneumothorax has been observed.

### Search results and characteristics of CP

Our literature search yielded a total 223 CPs of 217 cases in 111 reports in the PubMed database and 228 of 218 in 132 in the Japanese Ichushi database. Thirteen of the articles [4–16] reported 29 CPs in 22 juvenile cases. The mean age of all 451 CPs in 435 cases was 37.1 ± 6.6 years (range 13–55 years), with 414 right sided and 37 left sided. Among those, 374 had a diaphragmatic abnormality, such as endometrial implants or holes, while 77 did not.

### Laterality and diaphragmatic abnormality in JCP

**Fig. 2:**
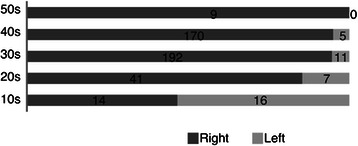
Laterality of catamenial pneumothorax in each generation

**Fig. 3:**
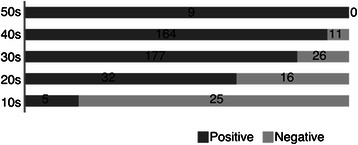
Diaphragmatic abnormalities in each generation

A total 465 CPs, 451 found in our literature search and 14 treated at our institution, were investigated. Among those, there were 30 cases of JCP (29 in literature search, 1 treated at our institution) and their characteristics are shown in Table 2. The laterality of pneumothorax in each is shown in Fig. 2. Left-sided pneumothorax was more often observed in younger patients, though JCP cases showed significantly less laterality as compared to UCP (p<0.0001). Diaphragmatic abnormalities reported are shown in Fig. 3. Fewer diaphragmatic lesions were observed in younger patients and JCP cases had a significantly lower incidence of diaphragmatic abnormalities as compared to UCP (p<0.0001). Many JCP cases had bullae/blebs as shown in Table 2.

**Table 2:** Juvenile catamenial pneumothorax

Case	Age	Laterality	Diaphragmatic lesion	Bellae/blebs	Literature
1	19	Left	-	-	Grevy [4]
2	18	Bilateral	-	+	Matsuge [5]*
3	18	Bilateral	-	+	Matsuge [5]*
4	13	Bilateral	-	+	Matsuge [5]*
5	18	Left	-	+	Matsuge [5]*
6	19	Left	-	-	Matsuge [5]*
7	18	Right	-	-	Matsuge [5]*
8	17	Left	-	-	Matsuge [5]*
9	17	Right	-	-	Matsuge [5]*
10	14	Right	+	+	Matsuge [5]*
11	17	Right	-	+	Matsuge [5]*
12	19	Right	+	-	Alifano [6]
13	16	Left	+	-	Ishikawa [7]
14	15	Bilateral	-	+	Shirai [8]
15	15	Left	-	+	Kashiwabara [9]
16	14	Left	+	+	Hiraoka [10]
17	16	Left	-	+	Ito [11]
18	17	Bilateral	-	+	Hamamoto [12]
19	19	Bilateral	-	+	Saito [13]
20	15	Right	-	-	Fatami [14]
21	19	Bilateral	-	+	Majak [15]
22	19	Right	+	-	Visouli [16]
23	18	Left	-	+	Present study

*Matsuge et al. reported a single case and cited 9

## Discussion

JCP patients showed quite different characteristics as compared to UCP in regard to laterality and diaphragmatic abnormalities. Since diaphragmatic lesions may be related to transdiaphragmatic migration, the pathogenesis of JCP might be different from that of UCP. In the present study, JCP patients as well as those in their 20s showed similar tendencies for those characteristics.

Three theories have been proposed to explain the pathogenesis of intrathoracic endometriosis; coelomic metaplasia, lymphatic or hematogenous embolization from the uterus or pelvis, and retrograde menstruation with subsequent transdiaphragmatic migration of endometrial tissue. However, none of those can adequately explain all of the clinical manifestations seen in CP cases. Kovaric et al. [17] reported autopsy findings showing that pleural and diaphragmatic lesions were always right-sided, whereas pulmonary parenchymal endometriosis usually had bilateral lesions. Cases with pleural and diaphragmatic lesions were explained by speculating transdiaphragmatic migration of endometrial tissue, while bilateral parenchymal lesions were speculated to be from hematogenous embolization, possibly coelomic metaplasia. In most cases of JCP, the pathogenesis may be related to hematogenous embolization or coelomic metaplasia.

Joseph and colleague [18] reported that the peak incidence of pelvic endometriosis occurred between 24 and 29 years of age, whereas that of thoracic endometriosis occurred approximately 5 years later. This difference may explain the time necessary for migration of endometrial tissue through the right diaphragm in UCP. On the other hand, transdiaphragmatic migration is difficult to explain when occurring in teenage cases, though hematogenous embolization or coelomic metaplasia may occur in younger patients. Since a similar tendency was observed in patients in their 20s, the pathogenesis of CP in younger individuals may be mainly related to hematogenous embolization of endometrial tissue, while that in older individuals might be mostly related to transdiaphragmatic migration.

Unlike abdominopelvic endometriosis, thoracic endometriosis is a rare condition that includes not only CP but also catamenial hemothorax, catamenial hemoptysis, and lung endometrial nodules. The etiological mechanisms of these diseases are not well understood and controversy remains. The present findings suggest that JCP might be a new entity, which would explain the different etiological mechanism from that seen with UCP.

## Conclusion

JCP cases showed characteristics different from those of UCP. Hematogenous embolization is likely related to JCP, while UCP might be explained by transdiaphragmatic migration.

## References

[1] Maurer CR, Schaal JA, Mendez FL (1958). Chronic recurring spontaneous pneumothorax due to endometriosis of the diaphragm. JAMA.

[2] Marshall M, Ahmed Z, Kucharczuc JC, Kaiser LR, Shrager JB (2005). Catamenial pneumothorax optimal hormonal and surgical management. Eur J Cardiothorac Surg.

[3] Alifano M, Trisolini R, Cancallieri A, Regnard JF (2006). Thoracic endometriosis: current knowledge. Ann Thorac Surg.

[4] Grevy C, Abdersen HJ, Hansen LG, Bloch AV (1987). Catamenial pneumothorax. Thorac Cardiovasc Surg.

[5] Matsuge S, Hosokawa Y, Sato K (2000). A case of catamenial pneumothorax in younger woman. Nikkyo.

[6] Alifano M (2003). Catamenial pneumothorax: a prospective study. Chest.

[7] Ishikawa N, Takizawa M, Yachi T, Hiranuma C, Sato H (2003). Catamenial pneumothorax in a young patient diagnosed by thoraciscopic surgery. Kyobu Geka.

[8] Shirai T, Oshima N, Miyagi N, Akamatsu H, Sunamori M (2003). A case of bilateral catamenial pneumothorax in 15 year-old girl. Nihon Kokyukigeka Gakkai Zasshi.

[9] Kashiwabara M, Itonaga K (2003). A case of catamenial pneumothorax diagnosed by endometrial tissue observed in resected bulla. Kikanshigaku.

[10] Hiraoka N, Odama S, Unohara K, Takano S, Taniai S, Shirai T (2005). Five cases of female pneumothorax with endometriosis in the bleb. Nihon Kokyuki Gakkai Zasshi.

[11] Ito Y, Urayama H (2006). A case of catamenial pneumothorax in young woman. Nihon Kokyukigeka Gakkai Zasshi.

[12] Hamamoto M, Takashita E, Okubo K (2006). Three cases of juvenile catamenial pneumothorax undergoing thoracoscopic surgery. Kikanshigaku.

[13] Saito T, Maniwa T, Kaneda H, Minami K, Sakaida N, Uemura Y (2010). Coexistence of catamenial pneumothorax and catamenial hemootysis in a patient with pulmonary hemangiomatosis-lisk foci: A case report. J Thorac Cardiovasc Surg.

[14] Fatimi SH, Khawaja R (2010). Catamenial Pneumothorax associated with ascites, eosinophilic pleural effusion, and relatively low glucose content. Am Surg.

[15] Majak P, Langebrekke A, Hagen OM, Qvigstad E (2011). Catamenial pneumothorax, clinical manifestations- a multidisciplinary challenge. Pneumonol Alergol.

[16] Visouli AN, Darwiche K, Mpakas A, Zarogoulidis P, Papagiannis A, Tsakiridis K (2012). Catamenial pneumothorax: a rare entity? Report of 5 cases and review of the literature. J Thorac Dis.

[17] Kovaric JL, Toll GD (1966). Thoracic endometriosis with recurrent spontaneous pneumothorax. JAMA.

[18] Joseph J, Sahn SA (1996). Thoracic endometriosis syndrome: new observations from an analysis of 110 cases. Am J Med.

